# An IL-18-centered inflammatory network as a biomarker for cerebral white matter injury

**DOI:** 10.1371/journal.pone.0227835

**Published:** 2020-01-24

**Authors:** Marie Altendahl, Pauline Maillard, Danielle Harvey, Devyn Cotter, Samantha Walters, Amy Wolf, Baljeet Singh, Visesha Kakarla, Ida Azizkhanian, Sunil A. Sheth, Guanxi Xiao, Emily Fox, Michelle You, Mei Leng, David Elashoff, Joel H. Kramer, Charlie Decarli, Fanny Elahi, Jason D. Hinman

**Affiliations:** 1 Memory & Aging Center, Department of Neurology, University of California San Francisco, San Francisco, CA, United States of America; 2 Department of Neurology and Center for Neurosciences, University of California, Davis, CA, United States of America; 3 Department of Public Health Sciences, University of California, Davis, CA, United States of America; 4 Department of Neurology, David Geffen School of Medicine, University of California Los Angeles, Los Angeles, CA, United States of America; 5 School of Medicine, New York Medical College, Vahalla, NY, United States of America; 6 University of Texas Health McGovern School of Medicine, Department of Neurology, Houston, TX, United States of America; 7 Department of Medicine Statistics Core, Department of Medicine, University of California Los Angeles, Los Angeles, CA, United States of America; 8 Department of Psychiatry, University of California San Francisco, San Francisco, CA, United States of America; University at Buffalo, UNITED STATES

## Abstract

Chronic systemic sterile inflammation is implicated in the pathogenesis of cerebrovascular disease and white matter injury. Non-invasive blood markers for risk stratification and dissection of inflammatory molecular substrates *in vivo* are lacking. We sought to identify whether an interconnected network of inflammatory biomarkers centered on IL-18 and all previously associated with white matter lesions could detect overt and antecedent white matter changes in two populations at risk for cerebral small vessel disease. In a cohort of 167 older adults (mean age: 76, SD 7.1, 83 females) that completed a cognitive battery, physical examination, and blood draw in parallel with MR imaging including DTI, we measured cerebral white matter hyperintensities (WMH) and free water (FW). Concurrently, serum levels of a biologic network of inflammation molecules including MPO, GDF-15, RAGE, ST2, IL-18, and MCP-1 were measured. The ability of a log-transformed population mean-adjusted inflammatory composite score (ICS) to associate with MR variables was demonstrated in an age and total intracranial volume adjusted model. In this cohort, ICS was significantly associated with WMH (β = 0.222, *p* = 0.013), FW (β = 0.3, *p* = 0.01), and with the number of vascular risk factor diagnoses (*r* = 0.36, *p*<0.001). In a second cohort of 131 subjects presenting for the evaluation of acute neurologic deficits concerning for stroke, we used serum levels of 11 inflammatory biomarkers in an unbiased principal component analysis which identified a single factor significantly associated with WMH. This single factor was strongly correlated with the six component ICS identified in the first cohort and was associated with WMH in a generalized linear regression model adjusted for age and gender (*p* = 0.027) but not acute stroke. A network of inflammatory molecules driven by IL-18 is associated with overt and antecedent white matter injury resulting from cerebrovascular disease and may be a promising peripheral biomarker for vascular white matter injury.

## Introduction

Cerebral small vessel disease (cSVD) is a leading contributor to vascular cognitive impairment and is estimated to cause 1/5^th^ of strokes in older adults [1]. cSVD has been associated with global cognitive decline, decreased executive function and reduced processing speeds [2–4]. Individuals with poorer cardiovascular health have higher cross-sectional cSVD burden and accelerated disease progression [5, 6]. Early detection of those at risk for cSVD would allow patients to improve their vascular health and possibly slow the progression of cSVD and cognitive decline associated with poor vascular health [7, 8].

Currently, diagnosis and risk stratification of cSVD relies on imaging techniques such as quantification of high signal intensities, or white matter hyperintensities (WMH) on T2-FLAIR imaging. In the context of cSVD, WMH are thought to be evidence of irreversible white matter injury with axonal and myelin damage [9, 10]. Recent studies using diffusion tensor imaging (DTI), found that DTI-derived measures, including fractional anisotropy (FA) and extracellular free water (FW), constitute sensitive biomarkers of early-stage white matter injury resulting from cSVD that occurs in advance of the lasting tissue injury measured by WMH [11–13]. However, MRI scans are costly and not indicated in the absence of neurologic symptoms, therefore limiting the ability to prevent or intervene in early cSVD to prevent cognitive decline late in life. Therefore, efforts are needed to identify tools that are both easily accessible and reproducible to facilitate earlier diagnosis and identification of patients at risk for cSVD.

At the cellular level, cSVD is hypothesized to result from endothelial dysfunction leading to subtle dysfunction of the blood brain barrier (BBB), resultant tissue damage, and progressive inflammatory responses within the brain [14–16]. High levels of chronic inflammation resulting from systemic vascular risk factors such as hypertension and diabetes are proposed to exacerbate cSVD by damaging cerebral endothelia. A number of systemic inflammatory indicators have been implicated in cSVD [17, 18], yet do not coalesce around a specific molecular pathway. In this study, we aimed to investigate the association of a biologically interconnected network of systemic inflammatory markers centered on the pleiotropic pro-inflammatory cytokine IL-18 with sCVD burden. IL-18 is associated with cardiovascular risk factors and disease [19–21], increases the expression of cell adhesion molecules on endothelial cells [22, 23], and may serve as a central coordinator for pathogenic inflammatory signaling [23]. Therefore, our investigation centered on IL-18 and associated proteins and their cross-sectional correlation with traditional and advanced neuroimaging measures of white matter integrity in a cross-sectional cohort design involving two populations at risk for cSVD. In a community-based aging population referred for cognitive evaluation, we used concurrent blood samples and MRI to develop a composite measure of inflammatory markers (IL-18, MPO, GDF-15, RAGE, ST2, and MCP-1) [18, 24–27] and correlated this inflammatory composite score (ICS) with MRI indicators of white matter injury. In a second cohort of acutely ill neurologic patients presenting for evaluation of stroke, we used an unbiased principal components analysis on a larger set of serum markers to derive inflammatory factors correlated with ICS to and test their association with cSVD as measured by Fazekas scoring of WMH as further validation of the ICS as a biomarker. Our findings suggest that an IL-18-centered network of systemic inflammation is associated with overt and antecedent white matter injury resulting from cerebrovascular disease and may be a promising biomarker of cSVD.

## Subjects/materials and methods

### MarkVCID study cohort

Research involving human subjects was approved by the Institutional Review Boards of the University of California, San Francisco (IRB #17–22314) and University of California, Davis (IRB #215830–47) and was conducted in compliance with the Health Information Portability and Accountability Act. One hundred and sixty-seven (167) community-dwelling older adults with normal cognition or mild cognitive impairment (MCI) were recruited from the University of California, San Francisco Memory and Aging Center or the Alzheimer’s Disease Center at University of California, Davis. Formal written consent including an estimation of capacity judged by study investigators was obtained and participants completed a baseline neuropsychological testing, neurological evaluation with a trained neurologist, Clinical Dementia Rate (CDR) completed with a study partner, and a blood draw. Blood samples were collected by peripheral vein venipuncture into serum-separating tubes, centrifuged immediately, processed for serum, aliquoted and stored at -80°C. One hundred and ten (110) study participants completed an MRI scan within six months of their baseline assessment, and a subset of 49 participants completed DTI. Participants were included in this study if they were considered non-demented by a formal consensus panel with a CDR total score of 0.0 or 0.5.

### ASPIRE study cohort

Research involving human subjects was approved by the Institutional Review Board of the University of California, Los Angeles (IRB # 14–001798) and was conducted in compliance with the Health Information Portability and Accountability Act. Formal written consent was obtained for all participants prior to the collection of blood samples. Capacity to provide consent was judged by study co-investigators based on the subject’s ability to articulate risks and benefits of participating after reviewing the consent form. Surrogate consent was approved by the IRB. Consecutive participants were patients presenting to the UCLA Emergency Department with symptoms concerning for stroke between December 2014 and June 2016 and offered to participate in the study. Study inclusion criteria were: onset of stroke symptoms within 8 hours of presentation (or within 2 hours of presentation if symptoms were present upon awakening); greater than 18 years of age and able to consent or had a suitable surrogate individual to consent on their behalf. Final clinical diagnosis was determined by a board-certified vascular neurologist. Blood samples were collected by peripheral vein venipuncture into heparin-containing tubes. Samples were kept on ice and then centrifuged immediately at 13,000 x g for five minutes at 4°C. The serum was collected and aliquoted into freezer vials for storage at -80°C. Subjects with evidence of CNS infection, known CNS malignancy, or recent head trauma as a potential cause of neurologic symptoms were excluded.

### Protein interactions

Tests for protein interactions among biomarkers was performed using the STRING database v11.0 (string-db.org) [28]. Multiple protein search tool was used to input GDF-15, MPO, ST2, IL-18, MCP-1, and RAGE. Settings for tests of interactions were: meaning of network edges = confidence; active interaction sources = all; minimum interaction score = medium confidence. A second shell of interactors limited to 5 was added for visual representation. Resulting analysis data were exported and are available via permanent web link (S1 File).

### Luminex assay and composite score generation

Serum levels of six markers of inflammation: myeloperoxidase (MPO), growth differentiation factor 15 (GDF-15), receptor for advanced glycation end products (RAGE), ST2, interleukin-18 (IL-18), and monocyte chemoattractant protein-1 (MCP-1) were measured in duplicate using a custom assay run across two plates on the Luminex platform (R&D Systems) measuring a total of 15 analytes: TNF-α, IL-6, ST2, MCP-1, RAGE, GDF-15, IL-18, CXCL5, CXCL6, IGFBP-2, MPO, ITGB3, BDNF, FGF-23, IL-17. The manufacturer protocol was followed and antigen binding within the assay was measured on a Luminex 200 System and analyzed using Milliplex Analyst 5.1. Four markers (TNF-α, BDNF, FGF-23, IL-17) were removed prior to analysis due to missing data and/or high proportions of values less than the limits of detection. Data points with coefficient of variance greater than 0.15 were excluded. To create a variable that measures inflammation of the whole network, we calculated an inflammation composite score (ICS) by normalizing raw inflammatory marker concentrations (pg/mL) using a log transformation, then standardizing data into z-scores, and finally taking the average of the z-scores across all six inflammatory markers. z-score generation was performed independently for each cohort.

### MarkVCID cohort MRI acquisition

Participants at the University of California, San Francisco completed MRI on a Siemens Trio 3T machine or Siemens Prisma 3T machine. T1, diffusion, and FLAIR sequences were collected. T1 acquisition: Volumetric MPRAGE sequences were used to acquire T1-weighted images of the entire brain (Sagittal slice orientation; slice thickness = 1.0 mm; slices per slab = 160; in-plane resolution = 1.0x1.0 mm; matrix = 240x256; TR = 2,300 ms; Trio: TE = 2.98 ms (Prisma: TE = 2.9); TI = 900 ms; flip angle = 9°). Diffusion (Trio) parameters: TR/TE 8200/86 ms; B  =  0 image and 64 directions at B  =  2000 s/mm2; FOV 220×220 mm2 and 2.2 mm thick slices; matrix 100×100 with 60 slices yielding 2.2 mm3 isotropic voxels / (TR/TE 8000/109 ms; B  =  0 image and 64 directions at B  =  2000 s/mm2; FOV 220×220 mm2 and 2.2 mm thick slices; matrix 100×100 with 55 slices yielding 2.2 mm3 isotropic voxels). Diffusion (Prisma) parameters: FOV 220×220 mm2 and 2.0 mm slice thickness; matrix 110×110 with 69 slices yielding 2.0 mm3 isotropic voxels; B  =  0 images with TR/TE 7080/72.20 ms; 96 directions at B  =  2500 s/mm2, 48 directions at B = 1000 s/mm2, and 30 directions at B = 500 s/mm all with TR/TE 2420/72.20 ms. FLAIR (Trio) parameters: slice thickness = 1.00mm; slices per slab = 160; in-plane resolution = 0.98x0.98mm; matrix = 256x256; TR = 6000ms; TE = 388ms; TI = 2100ms; flip angle = 120°. FLAIR (Prisma) parameters: slice thickness = 1.00mm; slices per slab = 176; in-plane resolution = 1.0x1.0mm; matrix = 256x256; TR = 5000ms; TE = 397ms; TI = 1800ms; flip angle = 120°.

All brain imaging at the University of California, Davis Imaging Research Center was performed on a 3T Siemens TIM Trio MRI System. Three sequences were used: an axial-oblique 3D T1 acquisition (FSPGR, TE: 2.9ms (min), TR: 2500ms (min), TI: 1100ms, flip angle: 7 degrees, slice thickness: 1mm, number of slices: 192, FOV: 256 x 256 mm, matrix size: 256 x 256, phase encoding direction: A/P), an axial-oblique 2D FLAIR Fast Spin Echo (TE: 90ms, TR: 9000ms, TI: 2500ms, flip Angle: 150 degrees, slice thickness: 1 mm interleaved, FOV: 256 x 256 mm, matrix size: 256 x 256, phase encoding direction: A/P, Options: Superior/Inferior saturation pulse On, 80 mm thick) and an axial-oblique 2D DTI sequence (Base sequence: Single-shot spin-echo echo planar imaging, TE: 101ms, TR: 9000ms, flip angle: 90 degrees, slice thickness: 2mm, FOV: 256 x 256 mm, matrix size: 128 x 128, phase encoding direction: P/A, Options: bandwidth: 1628Hz/Px, echo spacing: 0.7ms, EPI factor: 128). Diffusion weighted images were generated using gradients applied in 60 directions, with total gradient diffusion sensitivity measured at b = 1000 s/mm^2^, and 5 volumes with b = 0 s/mm2.

### ASPIRE cohort MRI acquisition

MRI was performed on either Siemens Avanto 1.5T or Siemens Trio 3T machines. Axial T_2_-weighted images were obtained continuously in 5-mm-thick sections with repetition time of 3800 milliseconds and time to echo of 116 milliseconds. The field of view was 220 mm, and the matrix was 384x384. Axial FLAIR images were obtained continuously in 5-mm-thick sections with repetition time of 9000 milliseconds and time to echo of 89 milliseconds. The field of view was 220 mm, and the matrix was 320x216. Axial diffusion-weighted images were obtained continuously in 5-mm-thick sections with repetition time of 5600 milliseconds and time to echo of 106 milliseconds. The field of view was 255 mm, and the matrix was 192x192.

### MarkVCID MRI processing

We used DTI measures of free water content (FW), FW-corrected fractional anisotropy (FA_COR_) and FW-corrected mean diffusivity (MD_COR_). Briefly, DTI images were first preprocessed using FSL software tools [29], including correction for eddy current-induced distortions and participant head movements. Individual uncorrected FA maps were co-registered to the FSL FA DTI template using linear and nonlinear transformations. Resulting transformation parameters were inversed and applied to the FSL white-matter labels atlas to provide a mask of WM region in the native DTI space of the individual. For each individual, overall measures of mean FW, FA_COR_ and MD_COR_ were computed by superimposing individual WM masks onto the respective individual DTI-derived maps and averaging values within these WM voxels. Segmentation of WMH, hippocampus and total cranial volume (TCV) were performed from FLAIR designed to enhance WMH segmentation [30] and T1-weighted images by automated procedures previously described and which demonstrates high inter-rater reliability [31–33]. For each individual, overall WMH burden and hippocampus volume were computed and normalized by TCV to account for differences in head volume. Resulting WMH burden was also log-transformed to normalize population variance.

### ASPIRE cohort Fazekas scoring

Two blinded authors (I.A. and V.K.) evaluated WMH on axial T_2_-weighted FLAIR images using the modified Fazekas rating scale to measure hyperintensity burden in periventricular and deep white matter regions [34, 35]. The total Fazekas score (FS) was obtained by summing the scores from periventricular and deep white matter regions and the average score used in subsequent analysis.

### Statistical analyses

All statistical analyses were conducted using SPSS (IBM Corp. Released 2013. IBM SPSS Statistics for Windows, Version 22.0. Armonk, NY: IBM Corp.) and SAS 9.4 (SAS Institute Inc.). Heat maps of ICS scores were generated in Prism (GraphPad). Means, standard deviations and frequencies are reported for the discovery and validation cohorts. Cohorts, including those with and without imaging, were compared using t-tests for continuous measures or Chi-square tests for categorical variables. Linear regression, controlling for age and total intracranial volume, was used to investigate the association of the inflammatory composite score (ICS) with measures of white matter integrity: WMH, FW, FA_COR_, and MD_COR_. Volumetric WMH were log-transformed prior to analysis to better meet the assumptions of the regression model. Standardized betas are reported. Correlation coefficients were estimated to assess the association between ICS and the total number of vascular risk factors. Principal component analysis was performed on the serum markers in the ASPIRE cohort to generate two main factors. Pearson correlations were calculated to assess the association of the principal components and the ICS score. Linear regressions models, controlling for age and gender, were used to evaluate the association of the serum principal components as well as the ICS itself on the outcome of Fazekas score.

## Results

### MarkVCID and ASPIRE cohort demographics

**Fig 1:**
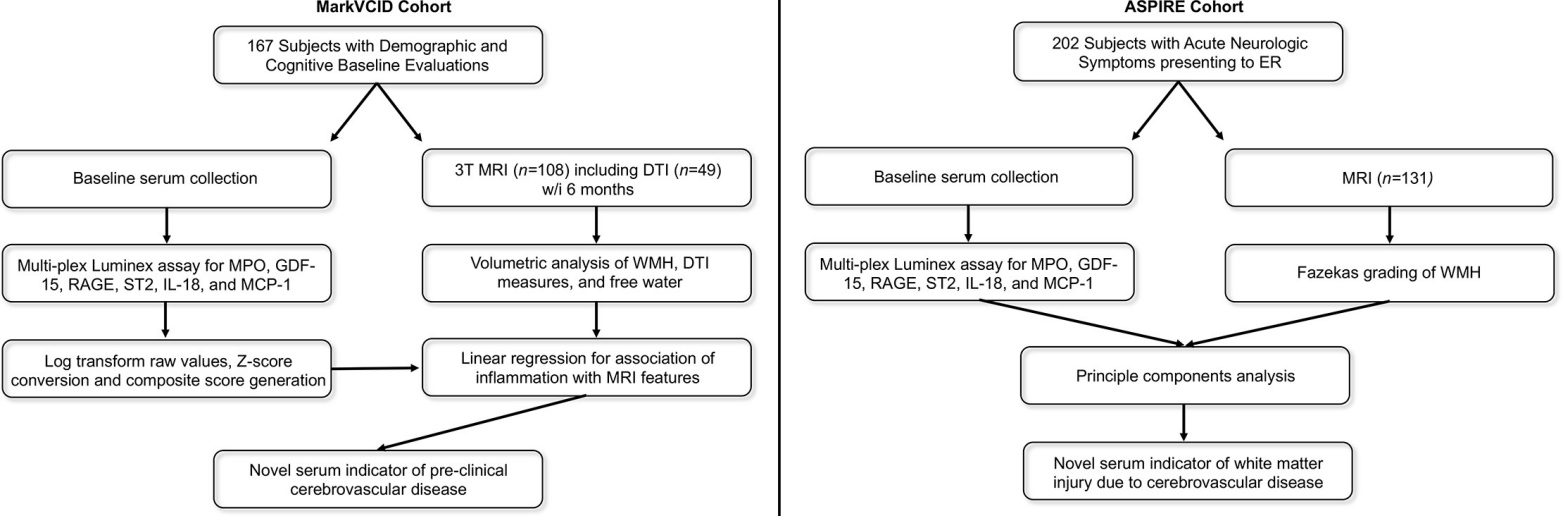
Imaging and fluid analysis workflows in the MarkVCID and ASPIRE cohorts. Workflow diagram of the MarkVCID cohort of 167 subjects that underwent detailed cognitive evaluations, MRI including DTI, and serum collections (left). Workflow diagram of the ASPIRE cohort of 202 subjects presenting with acute neurologic symptoms who underwent MRI and concurrent serum collection (right).

Fig 1 describes the subject identification, sample collection, and imaging workflows for each cohort. MarkVCID participants had a mean age of 76.4 ± 7.1 years, mean education of 15.3 ± 3.8 years, and 83 (49.7%) participants identified as female. All participants were functionally intact with 111 participants having a CDR total score of 0.0 and 56 participants having a CDR total score of 0.5. Participants had an average WMH volume (ml) of 6.94 ± 9.8, FW of 0.23 ± 0.03, FA_COR_ of 0.49 ± 0.08, and MD_COR_ of 0.54 ± 0.05. Overall, participants with brain imaging had better vascular and cognitive health (Table 1). ASPIRE participants had a mean age of 70.8 ± 1.2 years, 60 (45.8%) participants identified as female, and 10 (7.6%) participants had dementia. MarkVCID participants had an average ICS of 0.004 ± 0.56 and ASPIRE participants had an average ICS of 0.000 ± 0.60. Individual marker data is shown in Table 2. In the MarkVCID cohort, inflammatory markers measured in participants with T2-FLAIR imaging (n = 110) did not significantly differ from subjects without imaging. Participants with DTI (n = 49) did significantly differ from those without imaging in measures of GDF-15 (t = 2.6, *p* = 0.01) and IL-18 (t = 2.7, *p* = 0.007); participants with DTI had significantly lower levels of GDF-15 and IL-18. Additional raw imaging and serum inflammatory data are available upon request.

**Table 1:** MarkVCID and ASPIRE cohort demographics and vascular health history.

*MarkVCID Cohort*	Total	FLAIR	DTI	FLAIR vs. None	DTI vs. None	*ASPIRE Cohort*	Total
	n (%)			χ^2^ (*p*)	χ^2^ (*p*)		n (%)
*Total*	167 (100)	110 (65.8)	49 (29.3)			*Total*	131 (100)
*Gender (F)*	83 (49.7)	58 (52.7)	27 (55.1)	1.2 (0.33)	0.8 (0.40)	*Gender (F)*	60 (45.8)
*CDR = 0*	111 (66.5)	80 (72.7)	40 (81.6)	5.7 (0.024)*	7.2 (0.007)**	*CDR = 0*	*N/A*
*Dementia*	0 (0)	0 (0)	0 (0)			*Dementia*	*10 (7*.*63)*
*Stroke*	30 (18.2)	12 (11.1)	6 (12.2)	10.5 (0.001)**	1.6 (0.20)	*Stroke*	51 (38.9)
*MI*	13 (7.8)	6 (5.5)	4 (8.2)	2.4 (0.1)	0.01 (0.9)	*MI*	5 (3.8)
*AFib*	18 (10.8)	11 (10.0)	5 (10.2)	0.2 (0.6)	0.02 (0.9)	*AFib*	17 (13.0)
*HTN*	107 (64.1)	63 (57.3)	23 (46.9)	6.5 (0.01)*	8.84 (0.003)**	*HTN*	69 (52.7)
*HCL*	105 (64.0)	59 (54.6)	21 (43.7)	12.1 (0.001)**	12.1 (0.001)**	*HCL*	*45 (34*.*4)*
*Diabetes*	50 (30.3)	23 (21.1)	5 (10.4)	12.9 (0.001)**	12.7 (0.001)**	*Diabetes*	30 (22.9)
	Mean (SD)			t (*p*)	t (*p*)		Mean (SD)
*Age*	76.4 (7.1)	76.0 (6.9)	76.6 (7.0)	1.4 (0.2)	-0.24 (0.8)	Age	70.8 (1.2)
*Education*	15.3 (3.8)	15.6 (3.6)	16.3 (3.0)	-1.3 (0.1)	2.5 (0.01)*	Education	*N/A*
*BMI*	27.6 (5.6)	26.6 (5.3)	25.9 (4.9)	2.9 (0.004)**	2.6 (0.01)*	BMI	*N/A*
*Systolic BP*	139 (15.6)	138 (15.9)	135 (17.7)	1.6 (0.10)	2.3 (0.02)*	Systolic BP	159.0 (2.9)
*Diastolic BP*	72.3 (7.71)	72.9 (7.8)	73.4 (8.7)	-1.2 (0.2)	-1.1 (0.3)	Diastolic BP	86.5 (1.5)

Demographic and vascular health information for 167 MarkVCID Cohort participants and 131 ASPIRE Cohort participants. Chi squared and T-tests evaluated the group differences between MarkVCID Cohort participants with FLAIR imaging or DTI, and those without. Overall, participants with brain imaging had better vascular and cognitive health. Missing data for Mark VCID: Stroke (*n* = 2), HCL (*n* = 3), Diabetes (*n* = 2). F = Female, CDR = Clinical Dementia Rating, MI = Myocardial Infarction, AFib = Atrial Fibrillation, HTN = Hypertension, HCL = Hypercholesterolemia, BMI = Body Mass Index, BP = Blood Pressure

**Table 2:** MarkVCID and ASPIRE cohort inflammation levels.

*MarkVCID Cohort (n = 167)*		*ASPIRE Cohort (n = 131)*	
*Marker (pg/mL)*	Mean (SD)	*Marker (pg/mL)*	Mean (SD)
*IL-18*	363.4 (134.3)	*IL-18*	397.6 (211.2)
*ST2*	13146.1 (6995.1)	*ST2*	22113.2 (33073.9)
*MPO*	111567.7 (103133.0)	*MPO*	254458.16 (394569.63)
*MCP-1*	300.8 (161.9)	*MCP-1*	457.5 (222.1)
*GDF-15*	1887.8 (1764.4)	*GDF-15*	2758.4 (3575.5)
*RAGE*	1949.3 (958.9)	*RAGE*	2543.5 (1358.6)

Inflammatory marker data measured on 167 participants in the MarkVCID Cohort and 131 participants in the ASPIRE Cohort.

Although evaluated in different clinical settings, the MarkVCID and ASPIRE Cohorts had similar levels of vascular factors that increase the risk for cSVD. There were no statistical differences between the cohorts in gender (*p* = 0.50), history of myocardial infarction (*p* = 0.15), history of atrial fibrillation (*p* = 0.56), or history of diabetes (*p* = 0.17). As expected by participant enrollment procedures, the ASPIRE Cohort participants had more strokes (*p*<0.0001), and higher rates of dementia (*p* = 0.0003). The MarkVCID cohort was older in age (*p*<0.0001) and had a higher proportion of participants with hypertension (*p* = 0.047) and hypercholesterolemia (*p*<0.0001).

### Protein interactions

**Fig 2:**
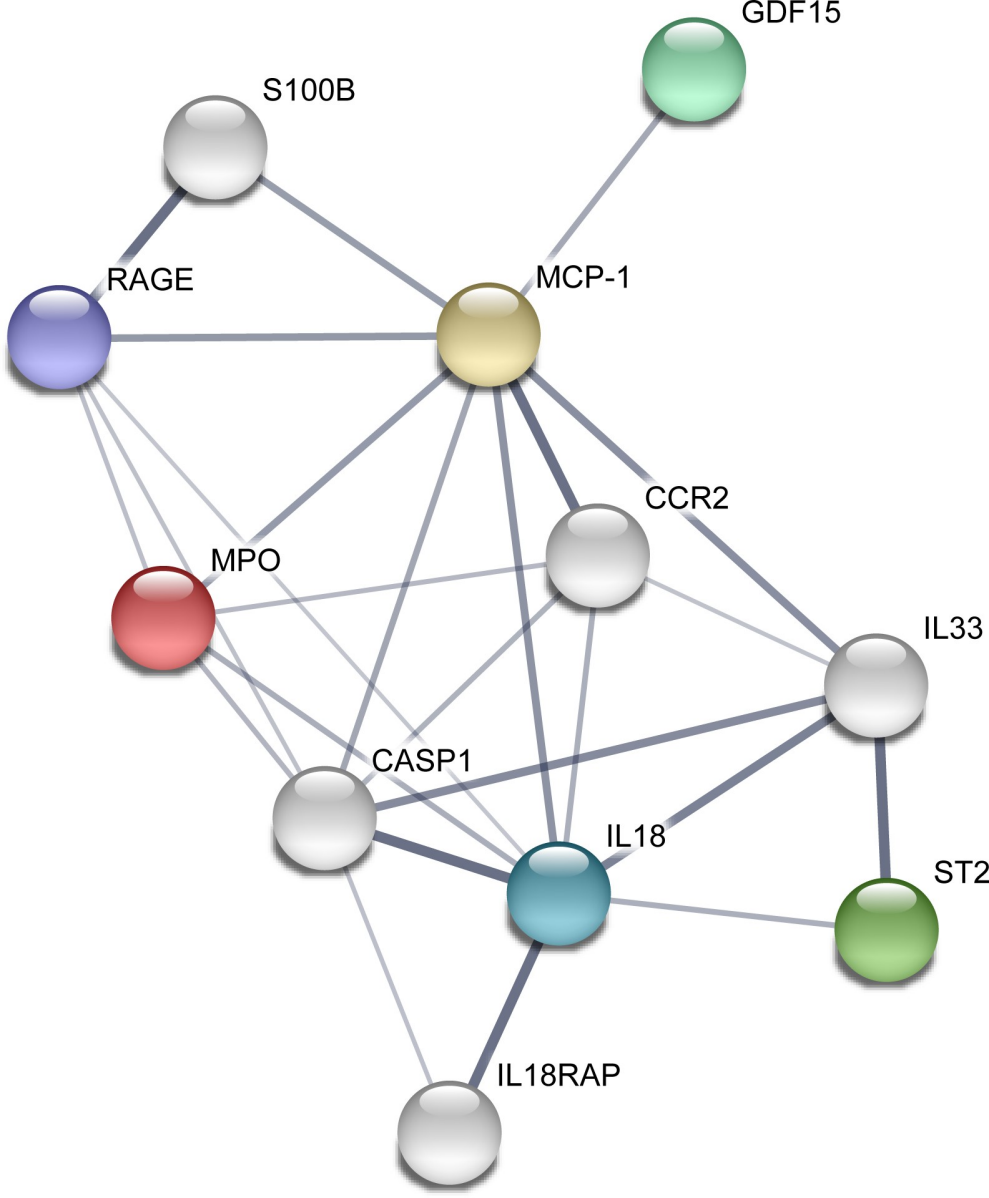
ICS components form an inflammatory network. STRING database query of the six ICS component analytes reveals a biologically interconnected network centered on IL-18 and highly related to inflammation (*p*-value for protein interactions = 0.00022). ICS component analytes are shown as colored nodes (bold) while first level interacting proteins are shown as white nodes. Line width reflects the strength of data support.

With independent evidence supporting a role for MPO, GDF-15, RAGE, ST2, IL-18, and MCP-1 in the development of white matter hyperintensities, we asked whether this group of validated biomarkers might be interconnected biologically. We performed STRING database analysis of these six components to determine if they function in a biologic network. Using the 6 validated protein biomarkers with a first shell of interactors, we identified a biologic network centered on IL-18 that was enriched for protein-protein interactions (*p* = 2.14x10^-8^) (Fig 2). This network was enriched for 75 gene ontology terms including positive regulation of leukocyte activation (GO.0002696, FDR = 0.00064); positive regulation of inflammatory response (GO.0050729, FDR = 0.0012); cytokine receptor binding (GO.0005126, FDR = 0.0015); cytokine activity (GO.0005125, FDR = 0.0015), and extracellular region (GO.0005576, FDR = 0.00029). The major signaling pathways center on interleukin signaling and IL-18 is the most connected node at the center of the network. Step-wise expansion of the network by known protein-protein interactions reveals a complex and tightly interconnected network that is strongly enriched for cytokine and immune system regulatory elements.

### Association of an IL-18 inflammatory network and white matter injury

**Fig 3:**
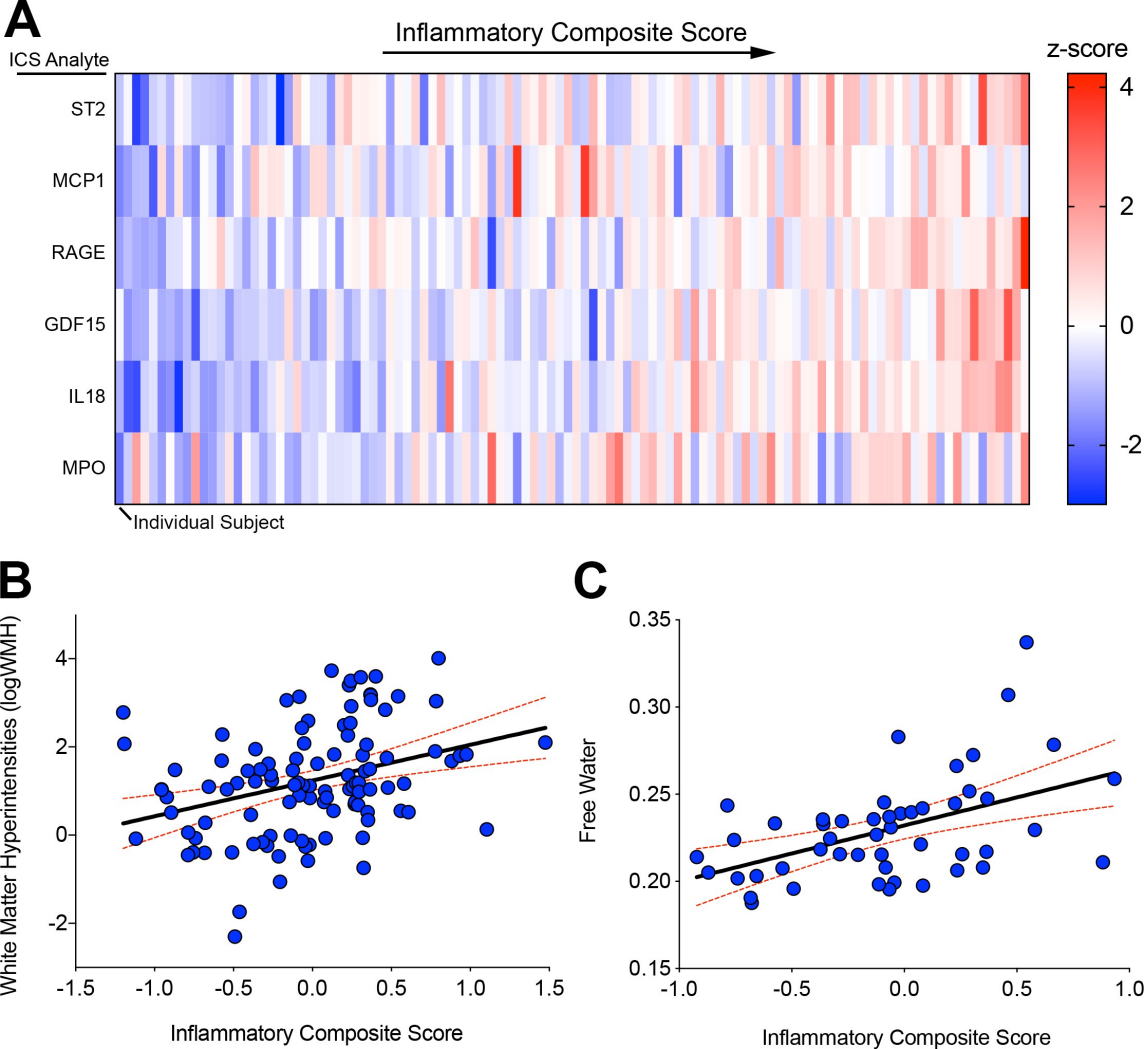
ICS correlates with MRI measures of cerebrovascular injury. Heatmap of z-scores for each of the individual analytes composing the ICS for each included subject in the MarkVCID cohort ordered left to right by ICS (average z-score of each analyte) (A). Scatter plot and regression line of logWMH vs. ICS (*n* = 110) (B). Scatter plot and regression line of free water vs. ICS (*n* = 49) (C). Red dashed lines indicate 95% confidence intervals.

**Fig 4:**
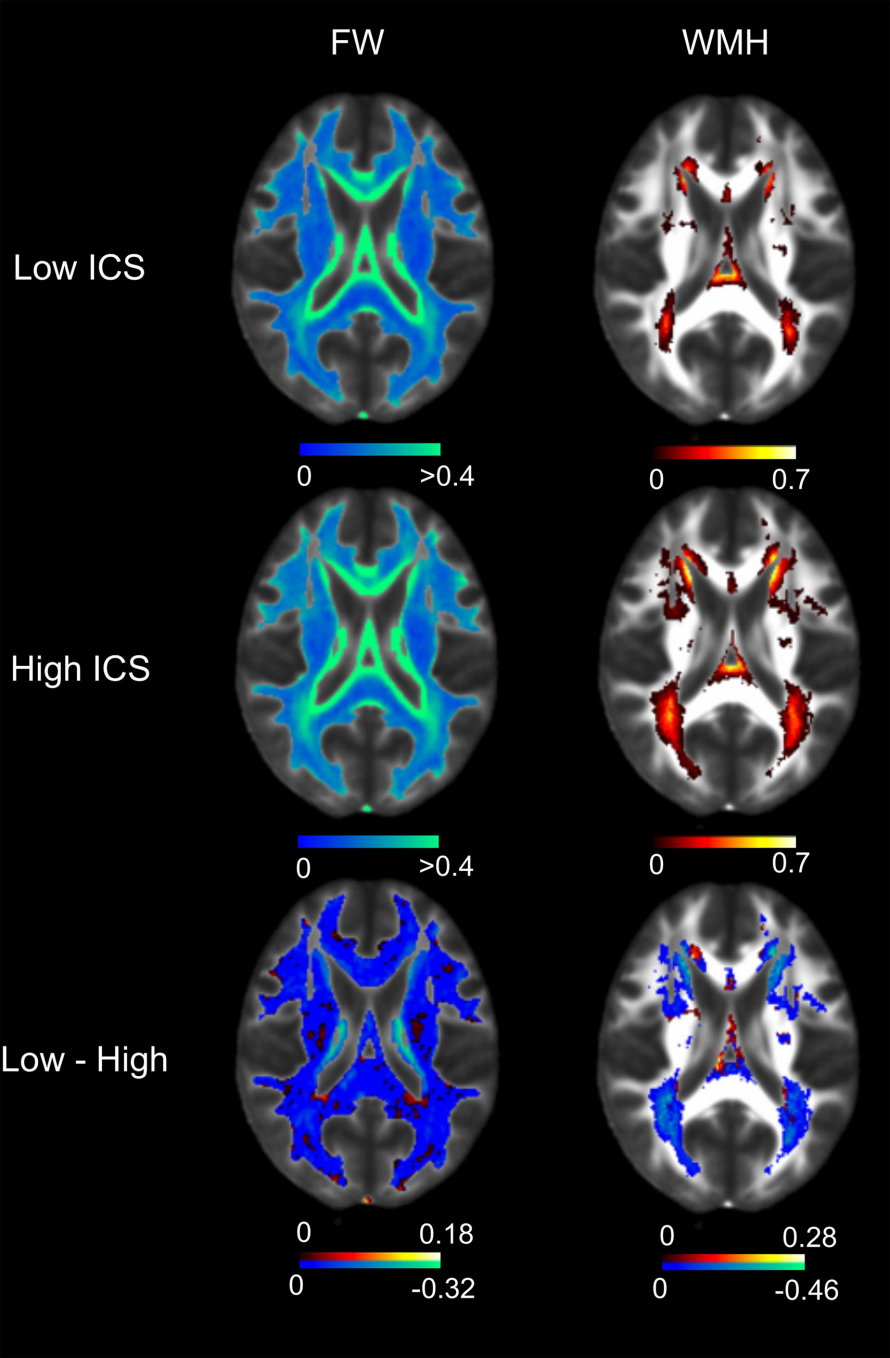
Association of ICS with overt and antecedent white matter injury. Average intensity maps of free water (FW) and frequency maps of white matter hyperintensities (WMH) of groups with low (upper) and high (middle) ICS groups dichotomized around median ICS. Lower panels illustrate voxel differences in FW and WMH between low and high ICS groups.

To determine how these interconnected biomarkers relate to vascular risk factors, white matter hyperintensities, and DTI measures of white matter injury, we used the means and standard deviations across the MarkVCID sample (n = 167) to create z-scores for each analyte for each participant. Z-scores for each analyte were averaged to generate an inflammatory composite score (ICS) for each subject. This approach reduces the impact of the relatively high population standard deviations common in biomarker studies. The z-scores for each ICS component analyte for each individual subject are shown in Fig 3A demonstrating that cumulative ICS was not driven by one outperforming analyte but rather reflect a true composite of the interacting inflammatory network. ICS was significantly associated with white matter hyperintensities (logWMH) (Fig 3B; β = 0.222, *p* = 0.013) as well as DTI FW (Fig 3C; β = 0.3, *p* = 0.01) but not with DTI FA_COR_ (β = 0.004, *p* = 0.98) or MD_COR_ (β = -0.2, *p* = 0.2). Spatial maps of average FW and WMH distributions on MRI in those subjects with low ICS (below median; upper panel) and high ICS (above median; middle panel) as well as the difference (lower panel) demonstrates the effect of high levels of IL-18 driven inflammation on subcortical white matter injury (Fig 4).

### The inflammatory composite score and vascular risk

**Fig 5:**
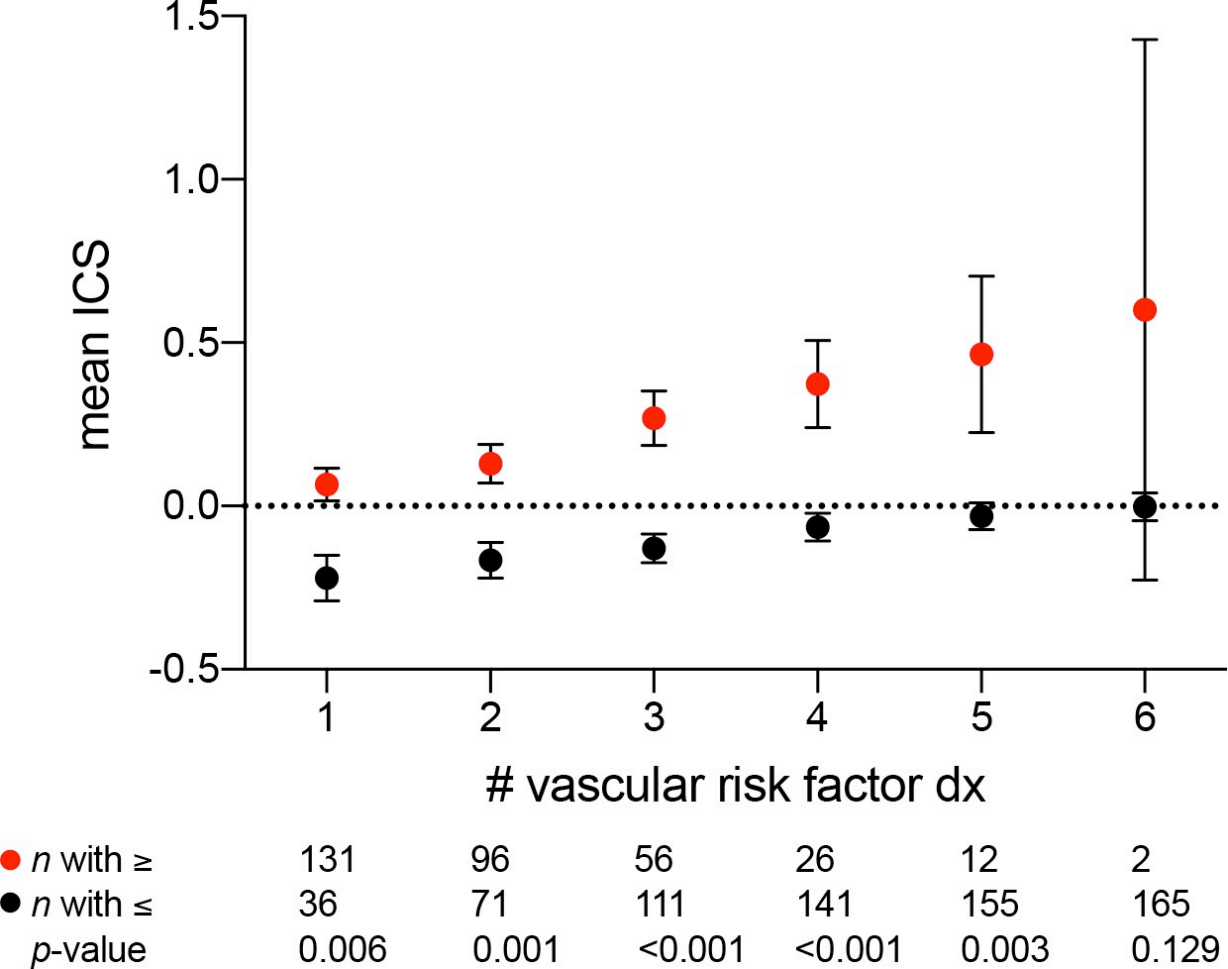
ICS increases with vascular risk factors. Mean ICS in groups with one or more vascular risk factor diagnoses (red) vs. those with less vascular risk factor diagnoses (black). All group comparisons were statistically significant at adjusted *p*<0.008 except between those with 6 vascular risk factor diagnoses (*n* = 2).

Vascular risk factors such as hypertension, hyperlipidemia, and diabetes increase the risk of developing white matter hyperintensities. Therefore, we reasoned that if ICS positively associates with white matter injury by MRI, then ICS should also scale with the burden of cardiovascular risk factors. The number of vascular risk factors significantly correlates with ICS (0.36, *p*<0.001). Categorization of the MarkVCID cohort by number of vascular risk factor diagnoses compared to those with fewer vascular risk factor diagnoses reveals a step-wise increase in mean ICS (Fig 5). Notably, the magnitude of the difference in mean ICS values increases as the number of vascular factors increases. Table 3 shows the group differences between MarkVCID cohort subjects with specific vascular risk factor diagnoses and those without.

**Table 3:** ICS associates with vascular risk factor diagnoses.

Vascular Risk Factor Diagnosis	Diff in Mean ICS	*p*-value
Atrial fibrillation (*n =* 18/167)	0.415	0.003
Stroke (*n =* 30/165)	0.427	0.000
Hypertension (*n =* 107/167)	0.253	0.004
Hyperlipidemia (*n =* 105/164)	0.209	0.021
Diabetes (*n =* 50/165)	0.399	<0.001
Myocardial infarction (*n =* 13/167)	0.146	0.364

Group differences between MarkVCID cohort subjects with specific vascular risk factor diagnoses. Missing data for Mark VCID: Hx Stroke (*n* = 2), Hx HCL (*n* = 3), Hx Diabetes (*n* = 2).

### Association of ICS with white matter injury in those at risk for stroke

**Fig 6:**
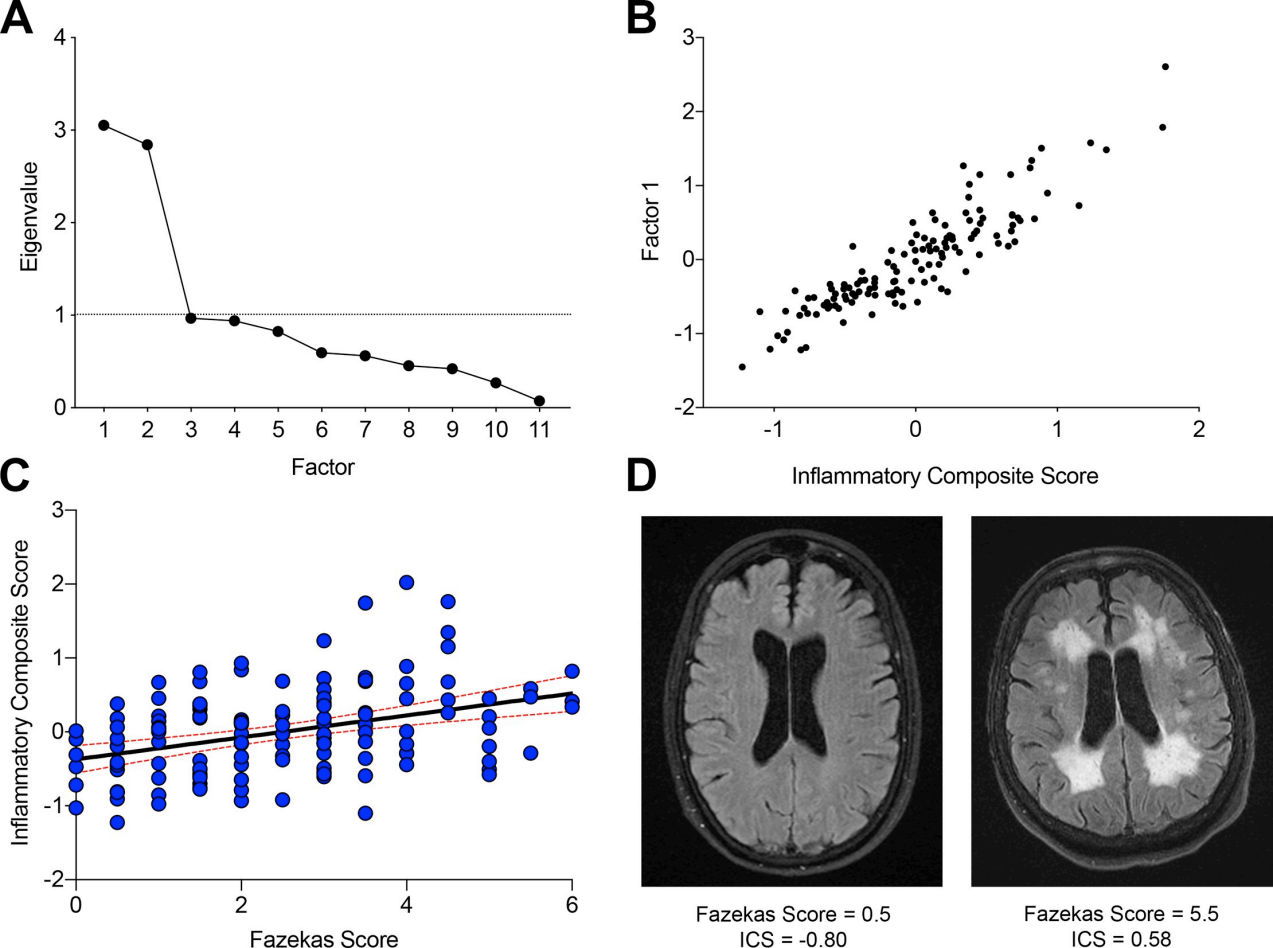
ICS is associated with white matter injury in those at risk for stroke. Scree plot of principal components analysis of data from ASPIRE cohort serum biomarker panel with two main factors (Factor 1 and Factor 2) driving variance (A). Scatter plot of Factor 1 values versus ICS in this cohort demonstrating a significant correlation (*r* = 0.94) (B). Scatter plot and regression line of modified Fazekas score and ICS for individual subjects (C). Red dashed lines indicate 95% confidence intervals. Representative T2/FLAIR MR images of ASPIRE subjects with low (left) or high (right) ICS scores (D).

To confirm the ability of ICS to detect WMH, we utilized serum samples from the ASPIRE biomarker study of acute stroke (*n* = 202), a single center study designed to identify acute biomarkers for ischemic stroke. Acutely obtained MRI scans (*n* = 168) were independently evaluated using the modified Fazekas scoring method. In those subjects with acutely obtained serum samples (*n =* 131), the average mean modified Fazekas score was 2.50 ± 1.53. Serum samples were assayed for biomarker levels and a principal component analysis was performed on 11 serum markers described in the methods section. This PCA identified two factors with eigenvalues >1 that account for 53% of the variance (Fig 6A). In this independent cohort, Factor 1 significantly correlated with ICS (*r* = 0.94, *p*<0.0001*)* (Fig 6B). The most significant contributors to Factor 1 were the log-normalized values of ST2, RAGE, GDF15, and IL-18, all core markers included in the ICS.

In an age- and gender-adjusted generalized linear regression model, the addition of Factor 1 significantly improved the detection of WMH as measured by the average modified Fazekas score in this cohort (*p* = 0.0267). Due to the strong correlation between Factor 1 and ICS in this cohort, we also assessed associations between ICS and WMH. In bi-variate analysis, ICS significantly correlated with average modified Fazekas score (*p*<0.0001) (Fig 6C) highly similar to the relationship of ICS with volumetric WMH in the MarkVCID cohort. In an age- and gender-adjusted model, the association between ICS and Fazekas score was *p* = 0.083. Representative FLAIR images from ASPIRE subjects with low and high ICS demonstrate the association with WMH (Fig 6D). Other demographic factors available in the ASPIRE cohort including stroke, hypertension, diabetes, and obesity were excluded from the model as they did not demonstrate significant effects on Fazekas score.

## Discussion

Cerebral small vessel disease contributes to dementia [36] and increases both the risk of stroke [37] and poor outcomes after stroke [38]. The progressive, silent development of cerebral small vessel disease necessitates the development of alternative methods for identifying those at risk. In population studies, mid-life vascular risk factors increase the risk of white matter injury resulting from cSVD [17]. However, the identification of biomarkers to assist in separating those with concurrent vascular risk factors yet no brain injury from those with vascular risk factors who already have evidence of pathology from cerebral small vessel disease is critical to developing therapeutic strategies to stem this growing public health challenge. Advances in imaging techniques such as DTI free water are one approach to detecting a higher risk population [12]. Reliable fluid-based biomarkers for early detection are another approach with certain advantages over imaging including accessibility, applicability, and the ability to test frequently enabling repeated measurements. Various proteomic and single molecule approaches for fluid biomarkers that associate with WMH have shown associations but lack an integrated conceptual framework that drives at disease pathogenesis. Here, we show that a biologically interconnected network of molecules reflecting a composite measure of inflammation is associated with T2/FLAIR white matter hyperintensities in both an aging community-based population and a population presenting for evaluation of acute neurologic symptoms. We also show that this composite measure of inflammation is associated with increases in DTI free water, further implicating an IL-18-centered inflammatory network in the disease process underlying cerebral small vessel disease. These data demonstrate a new, reproducible tool to identify those with and at risk for cSVD.

Existing data on purported serum and plasma biomarkers for cSVD strongly implicate inflammation in the cSVD disease process. In a study on 163 lacunar stroke patients and 183 hypertensive patients, patients with evidence of cSVD on brain MRI had significantly elevated levels of inflammatory markers: neopterin, sICAM-1 and sVCAM-1 [39]. Elevated levels of inflammation are associated with increased risk of major vascular events, infarct size, and death [14, 40, 41]. In the Framingham Study, men and women in the highest quartile of CRP levels at baseline had two to three times the risk of ischemic strokes compared to those in the lowest CRP quartile [42]. Similar increases in lacunar stroke risk were seen in subjects with elevated CRP in the SPS3 trial [43]. In this study, we selected MPO, GDF-15, RAGE, ST2, IL-18, and MCP-1 because research using each marker independently showed that the markers may be related to cSVD. Unlike previous studies, our team investigated the mechanistic relationship between MPO, GDF-15, RAGE, ST2, IL-18, and MCP-1 and discovered that the markers were related in a biological pathway. Via STRING database analysis, we identified a biologic network centered on IL-18.

IL-18 is a pleotropic pro-inflammatory cytokine implicated in multiple autoimmune disorders [44], vascular disease [19–21], acute stroke [45], and can be both generated and have action within the CNS [46]. Within the brain, IL-18 is largely produced by neurons [47, 48] but can also be found in infiltrating immune cells after ischemia [49, 50]. Here, we propose that the action of this IL-18 inflammatory network is to damage cerebral small vessels at the blood-brain barrier interface. The role this pathway plays in regulating downstream white matter injury resulting from IL-18-mediated cerebral vessel injury is unknown. The action of IL-18 is tightly regulated by IL-18 binding protein (IL-18BP) [51] and in autoimmune diseases, the serum IL-18/IL-18BP ratio is associated with disease severity [52–54]. Indeed, blocking the action of IL-18 using recombinant human IL-18BP (Tadekinig Alfa) is in late stage clinical trials for a number of autoimmune disorders [55, 56]. Future studies may consider measuring IL-18BP levels and/or targeting IL-18 as a novel therapeutic strategy for cerebral white matter injury.

By identifying the biologic connectivity of previously reported inflammatory cytokines and molecules, we begin to apply a more rigorous systems biology approach to the identification of reliable fluid biomarkers for cerebral small vessel disease. Harnessing this biologic connectivity provided a clear advantage in this study as evidenced by a strong correlation of ICS with the results of an unbiased principal components analysis (PCA) in a distinct cohort at increased risk for cSVD. By using PCA to identify an independent association of a collection of biomarkers (F1) that strongly correlates with our previously generated ICS in a different cohort, we functionally validate the use of the population mean-adjusted ICS to detect cSVD.

Systemic vascular risk factors have long been associated with increased inflammation that can be indirectly or directly measured [57]. High-sensitivity CRP (hsCRP) is the best example of an indirect inflammatory marker associated with vascular risk, white matter hyperintensities, and recurrent lacunar stroke. Vascular risk factors drive hsCRP levels upwards but provide no pathogenic clues to the underlying disease process and therefore require a large study population to verify their association with a disease outcome [58]. A number of inflammatory pathways with more direct signaling cascades have been associated with vascular risk factors including IL-18. Here we show evidence that a composite inflammatory measure (ICS) steadily increases as the number of vascular risk factors increases and that this associates with measures of silent cerebrovascular injury. Therefore, ICS could add to a clinical evaluation of stroke and dementia risk by providing a numerical severity to an individual subject’s cerebral microvascular injury and ongoing risk assessment [59]. Our cohorts lack sufficient data to determine the effect of vascular risk factor control on ICS. Presumably, sustained uncontrolled risk factors such as hypertension and diabetes would promote higher inflammatory composite scores however this remains to be determined. Precisely how these molecules damage the cerebral endothelia and promote the development of white matter injury is unknown. The present data implicate a signaling pathway centered on IL-18 as a potential driver of cerebral endothelia damage. Future studies can systemically test how persistent elevations of inflammatory cytokines directly damage cerebral endothelia and lead to white matter damage. Advances in isolating endothelial exosomes [2] will likely prove helpful to elucidate these mechanisms. Establishing the extent to which these silent changes may be reversible seems particularly critical to establish.

DTI free water is an emerging MR metric that indirectly measures leakage of extracellular fluid into white matter and precedes the development of T2/FLAIR white matter hyperintensities. DTI free water is associated with vascular risk factors including systolic blood pressure and arterial stiffness [11] and more recently has been shown to be associated with cognitive decline [13]. Exactly what DTI free water is measuring in tissue is unknown, however one hypothesis is that excess extracellular fluid results from blood-brain barrier leakage with leaking serum proteins damaging myelin and axons. Leakage of the blood-brain barrier is proposed to play a central role in the pathogenesis of cSVD with increased contrast-enhancement within T2/FLAIR white matter hyperintensities compared to normal appearing white matter using dynamic contrast enhanced MRI (DCE-MRI) techniques [60, 61]. In a population of recent lacunar stroke patients, blood-brain barrier leakage within white matter was also associated with impaired cognition at 1 year. The present study provides a mechanistic link between a cocktail of markers of peripheral inflammation and blood-brain barrier leakage in relationship to cSVD by demonstrating clear cross-sectional relationships between ICS and DTI free water. Further studies will be needed to establish causality between ICS and BBB leakage using longitudinal measures of fluid biomarkers and imaging with DCE-MRI.

In the presented imaging data from the MarkVCID cohort, we did not observe any clear regional pattern to the differences in either WMH or free water measures between those individuals with high or low ICS. This finding suggests that the observed association between WMH and free water with ICS is independent of differences in blood pressure and flow between anterior and posterior circulations. This result is not surprising given that the whole brain vasculature is exposed to elevated systemic inflammatory signals and whatever changes are induced are likely distributed globally throughout the brain. Notably, we also did not see any association between ICS and clinical stroke in the ASPIRE cohort, indicating that the elevated levels of inflammation measured by ICS are specifically linked to cSVD rather than an overall increased risk of cerebrovascular disease.

This study establishes a cross-sectional relationship between interconnected inflammatory molecules and MRI indicators of cerebral small vessel disease in two distinct populations. Limited to cross-sectional relationships, it does not firmly establish that IL-18-mediated inflammation is associated with cognitive decline nor with other conditions associated cSVD indicators, namely the risk of stroke and/or dementia. However, because ICS scales additively with increased vascular risk factors, which in turn are known to increase the risk of white matter hyperintensities and impaired cognition, we expect that longitudinal studies will demonstrate that ICS is predictive of longitudinal declines in cognition and/or the risk of future stroke. Additionally, we did not observe an association between ICS and other DTI metrics such as FA or MD. DTI free water and WMH are more directly linked on a continuum of white matter injury related to inflammation and blood-brain barrier leakage while FA and MD more directly reflect the integrity of axons within a functional tract. Moreover, the MarkVCID cohort is largely cognitive normal and with relatively healthy brains, and therefore lacks a wide range of FA/MD values. A further limitation of this study is the contrast in imaging methodologies used in the varying cohorts. The lack of precise volumetric assessment of white matter lesion volume in the ASPIRE cohort may underestimate the true burden of cSVD in this population. However, the ability of this set of inflammatory markers to retain its relationship with subjectively graded WMH in this population suggests that our approach in generating a population mean-adjusted composite inflammatory score may have broad generalizability as a biomarker for cSVD in at-risk with different risk factor profiles and demographics.

## Conclusion

Cerebral small vessel disease is provoked by cardiovascular risk factors through increased systemic sterile inflammation. This increase in systemic inflammation may be associated with a specific inflammatory pathway involving IL-18 signaling that can be targeted for therapeutic engagement. Fluid-based biomarkers to reliably identify those at risk for and with early indicators of cerebral small vessel disease resulting from inflammation can provide a widely accessible method for risk assessment, monitoring, and therapeutic development.

## Supporting information

S1 File Permanent weblink to STRING database results for ICS components. (DOCX)
