# Supplementary material for: An IL-18-centered inflammatory network as a biomarker for cerebral white matter injury
Source: PLoS One. 2020 Jan 24;15(1):e0227835. doi: 10.1371/journal.pone.0227835 (PMC6980497; doi:10.1371/journal.pone.0227835)
Supplement: S1 File — (DOCX) [file pone.0227835.s001.docx]

**Supplemental Information**:

An IL-18-centered inflammatory network as a biomarker for cerebral white matter injury

M. Altendahl^a^, P. Maillard^b^ , D. Harvey^c^, D. Cotter^a^ , S. Walters^a^, A. Wolf^a^, B. Singh^b^, V. Kakarla^d^, I. Azizkhanian^e^, S. A. Sheth^f^, G. Xiao^d^, E. Fox^a^, M. You^a^, M. Leng^g^, D Elashoff^g^, J. H. Kramer^a,h^, C. Decarli^b^, F. Elahi^a^, J. D. Hinman^d^

Permanent Weblink to String Database Results for ICS Components:

<https://version-11-0.string-db.org/cgi/network.pl?networkId=W9Nsc3UvYMOI>.
